# Ciliary neurotrophic factor has intrinsic and extrinsic roles in regulating B cell differentiation and bone structure

**DOI:** 10.1038/srep15529

**Published:** 2015-10-21

**Authors:** Maria Askmyr, Kirby E. White, Tanja Jovic, Hannah A. King, Julie M. Quach, Ana C. Maluenda, Emma K. Baker, Monique F. Smeets, Carl R. Walkley, Louise E. Purton

**Affiliations:** 1 Stem Cell Regulation Unit, St. Vincent’s Institute of Medical Research, Fitzroy, Vic. 3065, Australia; 2 Department of Clinical Genetics, Lund University, Lund, Sweden; 3 Department of Medicine at St. Vincent’s Hospital, The University of Melbourne, Fitzroy, Vic. 3065, Australia

## Abstract

The gp130 receptor and its binding partners play a central role in cytokine signalling. Ciliary neurotrophic factor (CNTF) is one of the cytokines that signals through the gp130 receptor complex. CNTF has previously been shown to be a negative regulator of trabecular bone remodelling and important for motor neuron development. Since haematopoietic cell maintenance and differentiation is dependent on the bone marrow (BM) microenvironment, where cells of the osteoblastic lineage are important regulators, we hypothesised that CNTF may also have important roles in regulating haematopoiesis. Analysis of haematopoietic parameters in male and female *Cntf*^−/−^ mice at 12 and 24 weeks of age revealed altered B lymphopoiesis. Strikingly, the B lymphocyte phenotype differed based on sex, age and also the BM microenvironment in which the B cells develop. When BM cells from wildtype mice were transplanted into *Cntf*^−/−^ mice, there were minimal effects on B lymphopoiesis or bone parameters. However, when *Cntf*^−/−^ BM cells were transplanted into a wildtype BM microenvironment, there were changes in both haematopoiesis and bone parameters. Our data reveal that haematopoietic cell-derived CNTF has roles in regulating BM B cell lymphopoiesis and both trabecular and cortical bone, the latter in a sex-dependent manner.

Cytokines that signal through the gp130 receptor play important roles in bone biology and neuronal survival^1^. One of these factors, ciliary neurotrophic factor (CNTF), binds with high affinity to a receptor complex consisting of the CNTF receptor (CNTFR), the gp130 receptor and leukemia inhibitory factor (LIF) receptor (LIFR). It can also bind with lower affinity to a complex consisting of the interleukin-6 receptor, GP130 and LIFR^2^.

Mice that lack *Cntfr* are perinatal lethal, surviving less than 24 hours after birth^3^. The *Cntfr*^−/−^ mice displayed significant loss of motor neurons and were unable to suckle, which likely resulted in their premature death^3^. In contrast, *Cntf*^−/−^ mice are viable, although they displayed significant reductions in motor neurons from 8 weeks of age^4^. Studies of the *Cntf*^−/−^ mice have also revealed that CNTF regulates normal bone structure in a sex-dependent manner^5^. While both male and female *Cntf*^−/−^ mice have shorter femur lengths compared to wildtype (WT) littermates, they showed different phenotypes in other bone parameters. Female *Cntf*^−/−^ mice had increased trabecular bone volume, caused by an increase in osteoblast number and bone formation rate. In contrast, male *Cntf*^−/−^ mice had normal trabecular bone but significantly reduced cortical bone^5^.

The bone marrow (BM) within the bone cavity is central for blood cell development. Structural or functional alterations to the BM microenvironment, which includes cells of the osteoblastic lineage^6^, have previously been shown to affect haematopoietic cell development. In particular, changes in osteoblast numbers have been associated with alterations in haematopoietic stem cell (HSC) numbers and function^7^,^8^.

B lymphopoiesis is also finely regulated by cells of the BM microenvironment. Tokoyoda *et al.* showed that B cell progenitors are in contact with stromal cells in the BM that express either chemokine (C-X-C motif) ligand 12 (CXCL12) or interleukin-7 (IL-7), two cytokines that are important for B cell differentiation^9^. Specifically, pre-pro B cells co-localised with CXCL12-expressing stromal cells while pro-B cells were in contact with IL-7-expressing stromal cells. Maturation beyond pro-B cells requires the developing B cells to migrate away from IL-7 and CXCL12-positive cells, and pre-B cells and immature IgM-expressing B cells were not found in contact with either of these cell types in the BM^9^.

Furthermore, *in vitro* culture studies showed that primary osteoblasts support B cell development^10^ and *in vivo* changes in osteoblast numbers altered the numbers of different subsets of B-lymphocytes^10^,^11^,^12^. However, the exact mechanism for the involvement of osteoblasts in the regulation of B cell development is not known. The level of osteoblasts does not correlate to the number of B cells, indicating that other, more complex mechanisms are involved^12^.

Despite the proven effect of CNTF on osteoblast numbers and function, nothing is known about the effects of CNTF on haematopoiesis. Here we have investigated the role of CNTF in haematopoiesis by analysing the haematopoietic cell phenotypes of *Cntf*^-−/−^ mice. Our analyses revealed that *Cntf*^−/−^ mice have altered B lymphopoiesis, however, the B cell phenotype differs based on sex, age and also the BM microenvironment in which the B cells develop.

## Results

### Female mice lacking Cntf have reductions of immature B cells in the bone marrow

As previously shown, 12-week-old *Cntf*^−/−^ mice have a sex-dependent bone phenotype, where female mice have increased bone volume accompanied by increased osteoblast numbers^5^. The lack of *Cntf* did not affect osteoclasts, and culture studies confirmed that CNTF directly inhibits osteoblast differentiation^5^. To investigate whether the bone phenotype was accompanied by changes to haematopoiesis, we analysed haematopoietic cell content in peripheral blood (PB), bone marrow (BM), spleen and thymus of female *Cntf*^−/−^ mice at 12 and 24 weeks of age. There were no significant differences in the numbers of leukocytes or erythrocytes in PB, BM or thymus, PB platelets, total body, or thymus weights (Supplementary Fig. 1). In contrast, spleen weights, spleen leukocytes, erythrocytes and platelets were significantly reduced in 12-week-old, but not 24-week-old, female *Cntf*^−/−^ mice (Supplementary Fig. 1).

**Figure 1:**
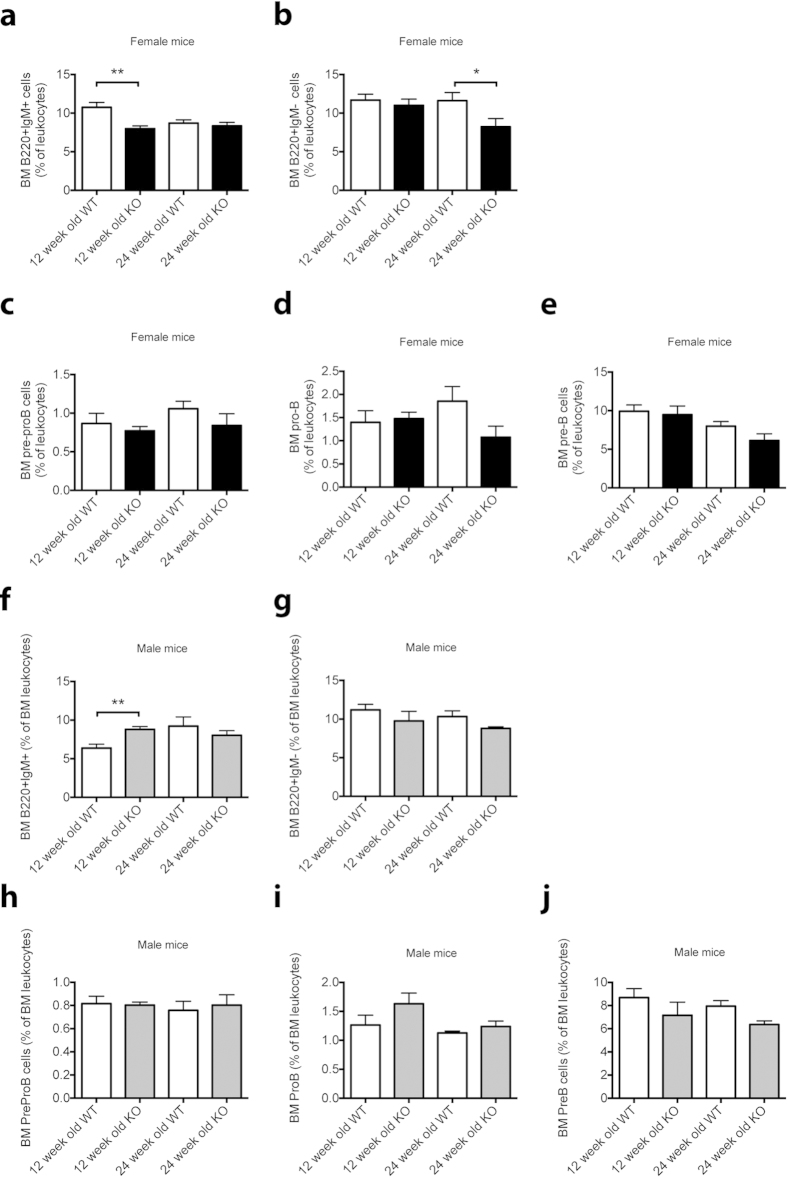
Analysis of BM B cell populations in female and male *Cntf*^−/−^ and *Cntf*^+/+^ mice. Shown are BM B cell populations in 12 and 24-week-old female (**a–e**) and male (**f–j**) *Cntf*^−/−^ (KO) and *Cntf*^+/+^ (WT) mice. The following populations were analysed: B220^+^IgM^+^ immature B cells (**a**,**f**), B220^+^IgM^−^ B cells (**b**,**g**), pre-pro B cells (**c**,**h**), pro-B cells (**d**,**i**) and pre-B cells (**e**,**j**). Data are shown as mean ± SEM, n = 3–9. The unpaired Student’s T-test was used for statistical comparisons between age- and sex-matched mice. *P < 0.05, **P < 0.01.

Interestingly, analysis of specific haematopoietic lineages using flow cytometry revealed an effect on B cell development in 12- and 24-week-old female *Cntf*^−/−^ mice, where we observed reduced proportions of immature B220^+^IgM^+^ B cells in the BM of 12-week-old *Cntf*^−/−^ mice and B220^+^IgM^−^ B cells in the BM of 24-week-old *Cntf*^−/−^ mice (Fig. 1a,b). However, although there were trends to reductions in pro-B cells in 24-week-old *Cntf*^−/−^ mice (*P* = 0.11 vs. 24-week-old *Cntf*^+/+^ females), BM progenitor B cells were not significantly affected (Fig. 1b–e). The decreases in the immature B cell populations in the BM were not reflected in PB or spleen (Supplementary Fig. 2). Furthermore, 12-week-old male *Cntf*^−/−^ mice had significantly increased B220^+^IgM^+^ cells in their BM (Fig. 1f). Aside from this, male *Cntf*^−/−^ mice at either 12 or 24 weeks of age did not have any obvious B cell phenotype (Fig. 1f–j and Supplementary Fig. 2). The effects on other haematopoietic lineages were modest in both female (Supplementary Fig. 3) and male (Supplementary Fig. 4) mice. Female mice displayed a slight disruption of T cell development as shown by an increase of CD8^+^ T cells in the spleen at 12 weeks (Supplementary Fig. 3) and a reduction of CD4^+^ T cells in the BM at 24 weeks (Supplementary Fig. 3).

**Figure 2:**
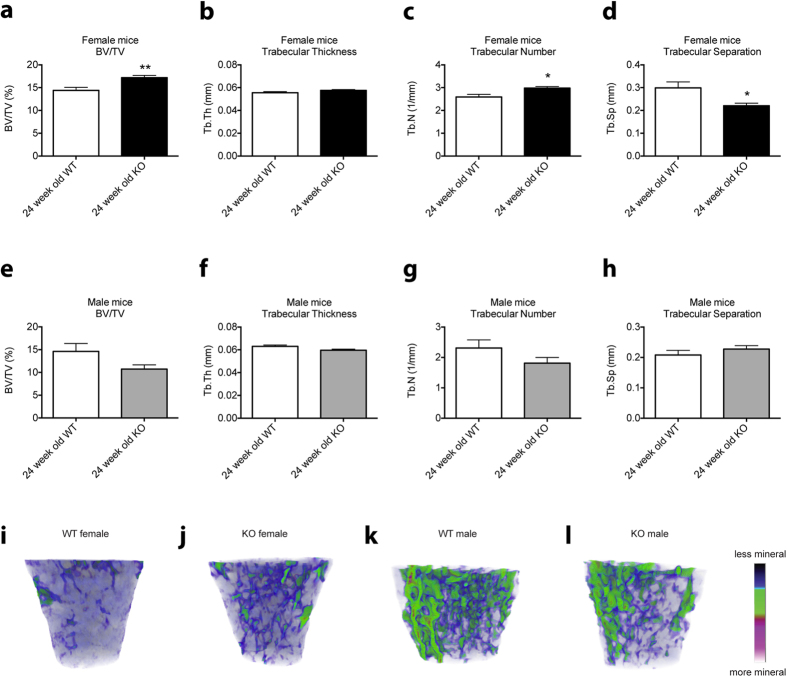
Analysis of trabecular bone in female and male *Cntf*^−/−^ and *Cntf*^+/+^ mice. The secondary spongiosa of the proximal femur was assessed by micro-computed tomography in femurs from 24-week-old female (**a–d,i,j**) and male (**e–h,k,l**) *Cntf*^−/−^ (KO) and *Cntf*^+/+^ (WT) mice. The following parameters were assessed: bone volume/total volume (BV/TV) (**a,e**), trabecular thickness (**b,f**), trabecular number (**c,g**) and trabecular separation (**d,h**). Representative micro-CT images of the femurs of (**i**) female *Cntf*^+/+^ mice or (**j**) female *Cntf*^−/−^ mice and femurs of (**k**) male *Cntf*^+/+^ mice or (**l**) male *Cntf*^−/−^ mice are shown at 24 weeks of age. Data are shown as mean ± SEM, n = 4–6. The unpaired Student’s T-test was used for statistical comparisons. *P < 0.05, **P < 0.01.

The trabecular and cortical bone phenotypes have previously been described for 12-week-old, but not 24-week-old mice. As shown in Fig. 2, 24-week-old female *Cntf*^−/−^ mice have increased trabecular bone volume, trabecular number and reduced trabecular separation (Fig. 2a–d,i,j), similar to that shown for 12-week-old female *Cntf*^−/−^ mice^5^. In contrast, and consistent with that observed in 12-week-old male *Cntf*^−/−^ mice, while the bone volume was slightly reduced in 24-week-old *Cntf*^−/−^ mice (*P* = 0.11 vs. *Cntf*^+/+^ males, Fig. 2e), trabecular bone parameters in 24-week-old male knock-out (KO) mice were not significantly affected by the loss of *Cntf* (Fig. 2e–h,k,l). Furthermore, the loss of *Cntf* did not affect cortical bone parameters in 24-week-old female or male mice (Supplementary Fig. 5 and 6), consistent with the phenotype observed in 12-week-old female *Cntf*^−/−^ mice, but different to that reported in 12-week-old male *Cntf*^−/−^ mice^5^.

### Il-7 expression is deregulated in Cntf^−/−^ female mice in both osteoblastic and pro-B cells

**Figure 3:**
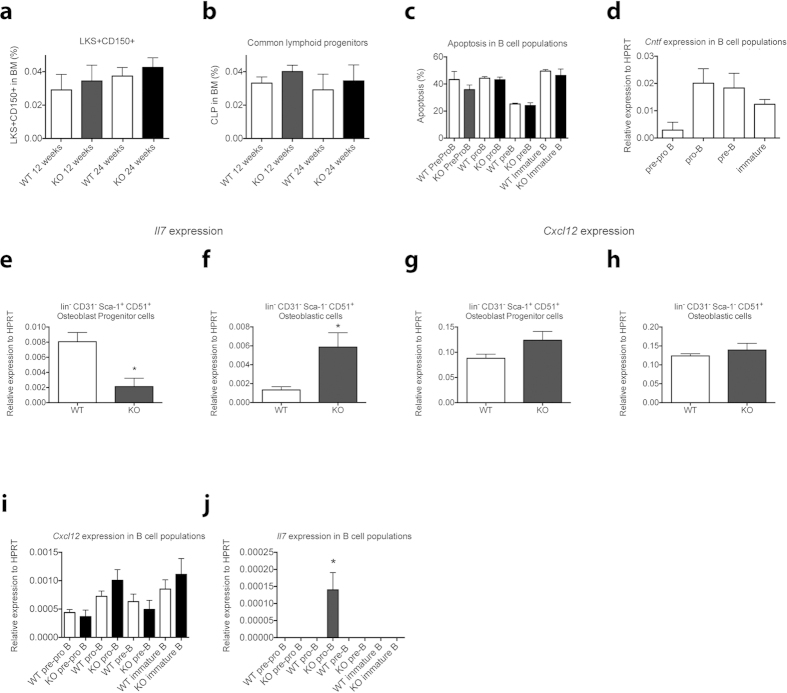
*Il7* and *Cxcl12* expression in sorted BM osteoblastic cells and B cell populations from 12-week- and 24-week-old female *Cntf*^−/−^ and *Cntf*^+/+^ mice. Proportions of (**a**) lineage negative, c-kit^+^, Sca-1^+^ CD150^+^ (LKS^+^CD150^+^) haematopoietic stem cells and (**b**) common lymphoid progenitors in BM. Analysis of apoptosis (**c**) and *Cntf* expression (**d**) in BM B cell populations from *Cntf*^−/−^ (KO) and *Cntf*^+/+^ (WT) 12-week-old mice. Osteoblast progenitors (**e,g**) and osteoblasts (**f,h**) were sorted from 12-week-old female *Cntf*^−/−^ (KO) and *Cntf*^+/+^ (WT) mice and the expression of *Il7* (**c,d**) and *Cxcl12* (**d,f**) was analysed (n = 3 separate sort experiments but within each experiment, 3–4 mice were pooled). BM was also sorted into B cell populations and analysed for the expression of *Cxcl12* (**i**) and *Il7* (**j**). Data are shown as mean ± SEM, n = 3–4. One-way analysis of variance followed by post-hoc testing or the unpaired Student’s T-test was used for statistical comparisons. *P < 0.05.

To determine the potential mechanisms resulting in reduced B cell populations in the BM of female *Cntf*^−/−^ mice we performed further studies using female *Cntf*^+/+^ and *Cntf*^−/−^ mice. There were no differences in the proportions of long-term repopulating haematopoietic stem cells [lineage negative, c-kit^+^, Sca-1^+^ (LKS^+^) CD150^+^ cells] or common lymphoid progenitor cells (CLPs) in the BM of the 12- or 24-week-old female *Cntf*^−/−^ mice (Fig. 3a,b). Furthermore, the LKS+ CD150- cells consisting of short-term repopulating stem cells and multipotent progenitors were not altered in the *Cntf*^−/−^ mice compared to their wildtype littermates [LKS^+^CD150^-^ cells (% of total BM, mean ± SEM, n = 4–5 mice): *Cntf*^+/+^: 0.139 ± 0.026%; *Cntf*^−/−^: 0.107 ± 0.030%]. We next analysed the level of apoptosis in B cell populations in 12-week-old females but could not detect any changes that could explain the reduction of the immature B cells (Fig. 3c). We determined that *Cntf* was expressed by developing B lymphocytes sorted from WT BM, especially by pro-B, pre-B and immature B220^+^IgM^+^ B cells (Fig. 3d), suggesting potential intrinsic roles for CNTF in regulating B lymphopoiesis. In contrast, none of the B cell populations expressed CNTFR (data not shown).

Osteoblasts have previously been shown to express CNTFR, but not CNTF^5^. To determine if there might be extrinsic changes in the BM microenvironment supporting B lymphopoiesis, we first examined the expression of B cell regulatory factors in whole BM obtained from 12-week-old female *Cntf*^−/−^ and *Cntf*^-+/+^ mice, focusing on extrinsic factors that have been shown to be essential in regulating B lymphopoiesis^13^. There were no changes in the expression of *Flt3 ligand*, or *Rankl (receptor activator of nuclear factor-κβ ligand)* observed in whole bone marrow mRNA obtained from *Cntf*^−/−^ or *Cntf*^+/+^ mice (data not shown). In contrast, we observed trends to increased expression of *Il7* and *Cxcl12* transcripts in BM from 12-week-old female *Cntf*^−/−^ compared to *Cntf*^+/+^ mice (data not shown). We therefore purified osteoblast progenitors (lineage, CD45 and CD31 negative, Sca-1^+^, CD51^+^) and relatively mature osteoblast cell (lineage, CD45 and CD31 negative, Sca-1 negative, CD51^+^) populations from 12-week-old female *Cntf*^−/−^ and *Cntf*^+/+^ mice using fluorescence activated cell sorting (FACS)^14^,^15^ and analysed the expression of *Il7* and *Cxcl12* in these populations. The expression of *Il7* was significantly reduced in osteoblast progenitors (Fig. 3e) and increased in osteoblasts sorted from *Cntf*^−/−^ mice (Fig. 3f). In contrast, the expression of *Cxcl12* was unchanged in both populations (Fig. 3g,h). We also sorted B cell populations from the same mice and analysed the expression levels of *Cxcl12* and *Il7. Cxcl12* expression was very low and unchanged in B cells isolated from *Cntf*^−/−^ mice compared to *Cntf*^+/+^ mice (Fig. 3i). Intriguingly, while the different B cell populations sorted from *Cntf*^+/+^ mice had undetectable expression of *Il7* (Fig. 3j), we detected *Il7* expression exclusively in pro-B cells sorted from *Cntf*^−/−^ mice (Fig. 3j).

### Deregulated B cell differentiation and altered bone parameters observed in Cntf^−/−^ mice are partly due to haematopoietic cell-intrinsic effects on Cntf in haematopoietic cells

The mice used for this study are complete knockouts, hence *Cntf* expression is lacking from all cells. The effects observed in B cell development could thus be a result of indirect stimulation from the surrounding microenvironment or from intrinsic effects in the haematopoietic system. To delineate whether the observed changes are intrinsic to the haematopoietic cells or whether they are induced by the microenvironment, we transplanted either *Cntf*^+/+^ or *Cntf*^−/−^ BM cells into WT mice or WT BM cells into *Cntf*^+/+^ or *Cntf*^−/−^ mice. Given the differences observed between the female and male sexes, we performed female into female and male into male BM transplants.

**Figure 4:**
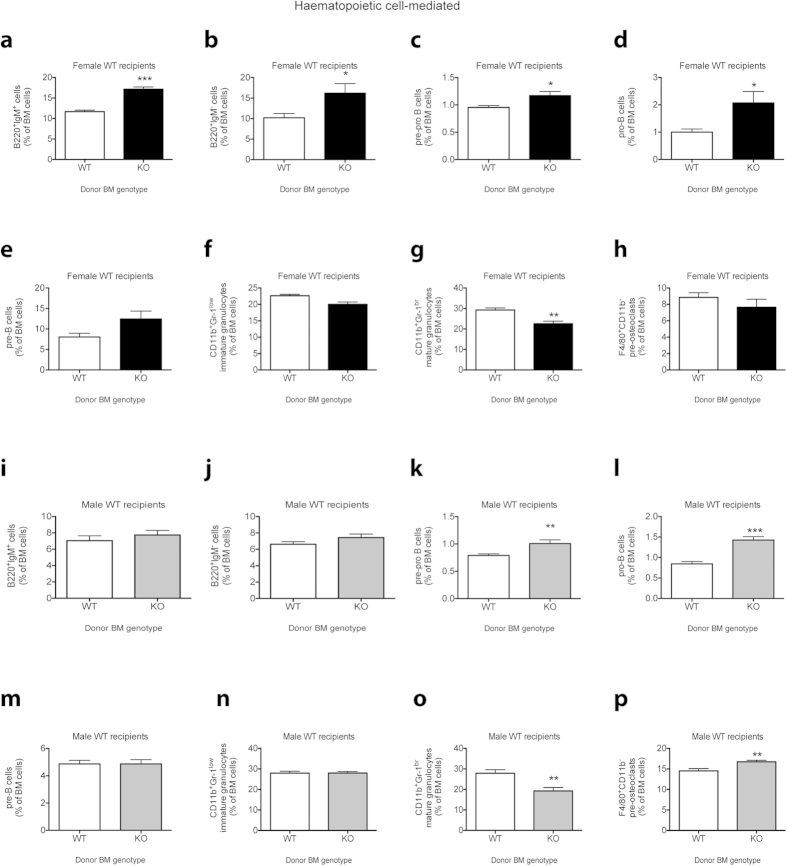
Haematopoietic lineage analysis of BM from WT mice following transplantation of *Cntf*^−/−^ and *Cntf*^+/+^ BM. Female (**a–h**) or male WT mice (**i–p**) were transplanted with *Cntf*^−/−^ (KO) and *Cntf*^+/+^ (WT) BM cells (female cells into female mice and male cells into male mice) and the BM content was analysed using FACS at 12 weeks post-transplantation. Presented here is the analysis of B220^+^IgM^+^ immature B cells (**a,i**), B220^+^IgM^-^ B cells (**b,j**), pre-pro B cells (**c,k**), pro-B cells (**d,l**), pre-B cells (**e,m**), CD11b^+^Gr-1^low^ immature granulocytes (**f,n**), CD11b^+^Gr-1^br^ mature granulocytes (**g,o**) and F4/80^+^CD11b^−^ pre-osteoclasts (**h,p**). Data are shown as mean ± SEM, n = 5–10. The unpaired Student’s T-test was used for statistical comparisons. *P < 0.05, **P < 0.01, *** *P* < 0.005.

There were no changes in PB, BM, spleen or thymus cellularities or spleen or thymus weights in WT recipients of *Cntf*^−/−^ BM cells of either sex (Supplementary Fig. 7). However, transplantation of female *Cntf*^−/−^ BM cells into a female WT microenvironment resulted in a more profound, but intriguingly different, haematopoietic phenotype than that observed in full *Cntf*^−/−^ female mice. There were significant increases in immature B220^+^IgM^+^ B cells (Fig. 4a) and B220^+^IgM^−^ B cells (Fig. 4b). Fractionation of the B220^+^IgM^-^ cells revealed significant increases in pre-pro and pro-B cell populations (Fig. 4c,d). Furthermore, a trend towards an increase in pre-B cells was observed (Fig. 4e). Analysis of myeloid cells in the BM (Fig. 4f–h) showed a significant reduction of mature granulocytes (Fig. 4g). No other changes were observed in any of the haematopoietic cells in the female WT recipients (Fig. 4f,h and data not shown).

Similar haematopoietic changes were observed in male WT recipients transplanted with male *Cntf*^−/−^ BM cells (Fig. 4i–p). These recipients also had significantly increased pre-pro B and pro-B cells (Fig. 4k,l). Furthermore, and also similar to the female WT recipients, the male WT recipients of *Cntf*^−/−^ BM cells had significant reductions in mature granulocytes (Fig. 4o) in their BM. Furthermore, the proportions of F4/80^+^CD11b^−^ pre-osteoclasts were increased in the BM of the WT male recipients (Fig. 4p).

**Figure 5:**
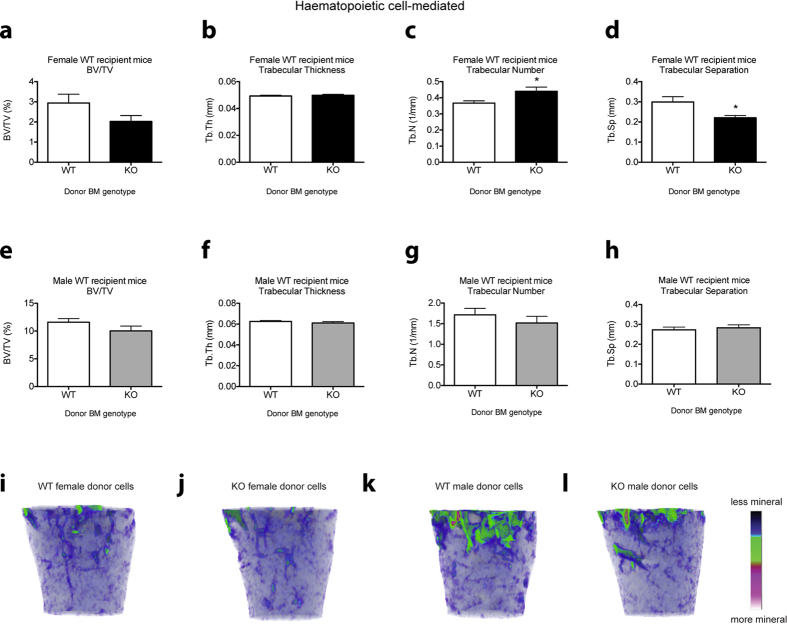
Trabecular bone phenotype in female and male WT recipients of *Cntf*^−/−^ and *Cntf*^+/+^ BM. Female (**a–d**) or male WT mice (**e–j**) were transplanted with *Cntf*^−/−^ (KO) and *Cntf*^+/+^ (WT) BM cells (female cells into female mice and male cells into male mice) and femurs were analysed using micro-computed tomography at 12 weeks post-transplantation. Shown are the trabecular bone parameters: bone volume/total volume (BV/TV) (**a,e**), trabecular thickness (**b,f**), trabecular number (**c,g**) and trabecular separation (**d,h**). Representative micro-CT images of the femurs of WT female mice transplanted with (**i**) female *Cntf*^+/+^ BM or (**j**) female *Cntf*^−/−^ BM and femurs of WT male mice transplanted with (**k**) male *Cntf*^+/+^ BM or (**l**) male *Cntf*^−/−^ BM are shown at 12 weeks post-transplant. Data are shown as mean ± SEM, n = 5–10. The unpaired Student’s T-test was used for statistical comparisons. *P < 0.05.

There were also significant differences observed in the female and male bone parameters when *Cntf*^−/−^ BM cells were transplanted into WT recipients. In female recipients, there was a trend to decreased trabecular bone volume (Fig. 5a), no changes to trabecular thickness (Fig. 5b), significant increases in trabecular number (Fig. 5c) and significant reductions in trabecular separation (Fig. 5d) in WT recipients transplanted with *Cntf*^−/−^ BM cells (Fig. 5j) compared to WT recipients transplanted with *Cntf*^-+/+^ BM cells (Fig. 5i). These phenotypes were similar to those observed in 12-week^5^ and 24-week-old female *Cntf*^−/−^ mice (Fig. 2). In contrast, and also the same as that observed in 12-week and 24-week-old female *Cntf*^−/−^ mice, female WT recipients transplanted with female *Cntf*^−/−^ BM cells had no changes in cortical bone parameters (Supplementary Figs 6 and 8).

The bone phenotypes observed in *Cntf*^−/−^ male mice were also recapitulated in male WT recipients of *Cntf*^−/−^ BM cells. These recipients had no changes in trabecular bone parameters (Fig. 5e–h,k,l), but did have significant reductions in cortical bone parameters (Supplementary Figs 6 and 8). Similar to the phenotypes observed in 12-week-old male *Cntf*^−/−^ mice^5^, WT male recipients of male *Cntf*^−/−^ BM cells had significant reductions in endocortical and periosteal perimeters (Supplementary Figs 6 and 8). There were slight but not significant reductions in cortical thickness (Supplementary Figs 6 and 8), significant reductions in mean polar movement of inertia, cortical area and marrow area (Supplementary Figs 6 and 8).

**Figure 6:**
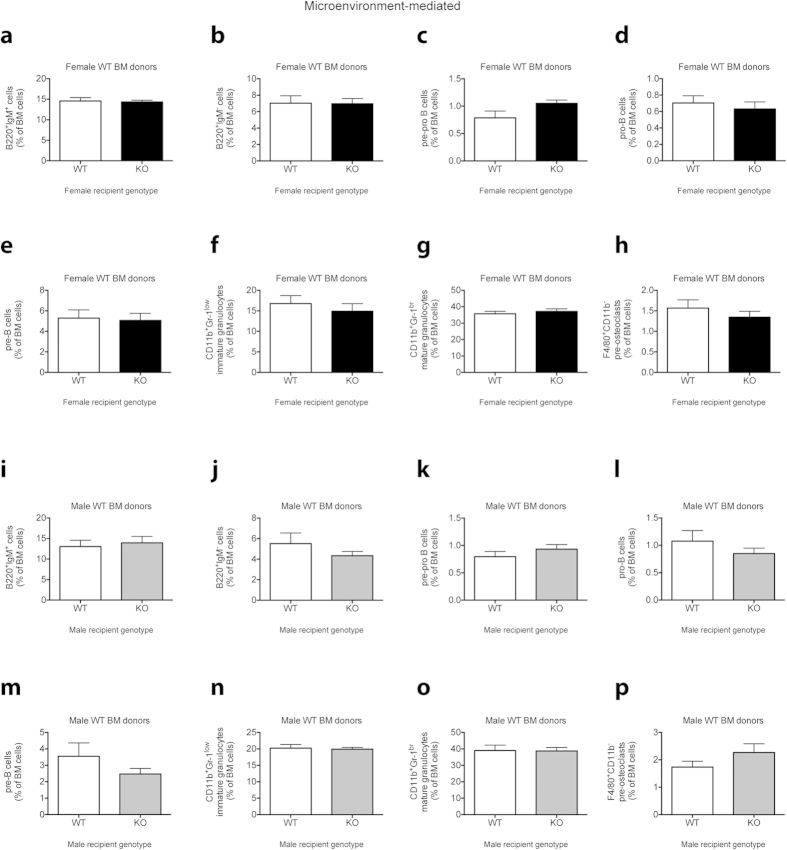
Haematopoietic lineage analysis of BM from *Cntf*^−/−^ and *Cntf*^+/+^ mice following transplantation of WT BM. Female (**a–h**) or male *Cntf*^−/−^ (KO) and *Cntf*^+/+^ (WT) mice (**i–p**) were transplanted with WT BM cells (female cells into female mice and male cells into male mice) and the BM content was analysed using flow cytometry at 12 weeks post-transplantation. Lineage analysis shows B220^+^IgM^+^ immature B cells (**a,i**), B220^+^IgM^-^ B cells (**b,j**), pre-pro B cells (**c,k**), pro-B cells (**d,l**), pre-B cells (**e,m**), CD11b^+^Gr-1^low^ immature granulocytes (**f,n**), CD11b^+^Gr-1^br^ mature granulocytes (**g,o**) and F4/80^+^CD11b^−^ pre-osteoclasts (**h,p**). Data are shown as mean ± SEM, n = 5–6. The unpaired Student’s T-test was used for statistical comparisons.

**Figure 7:**
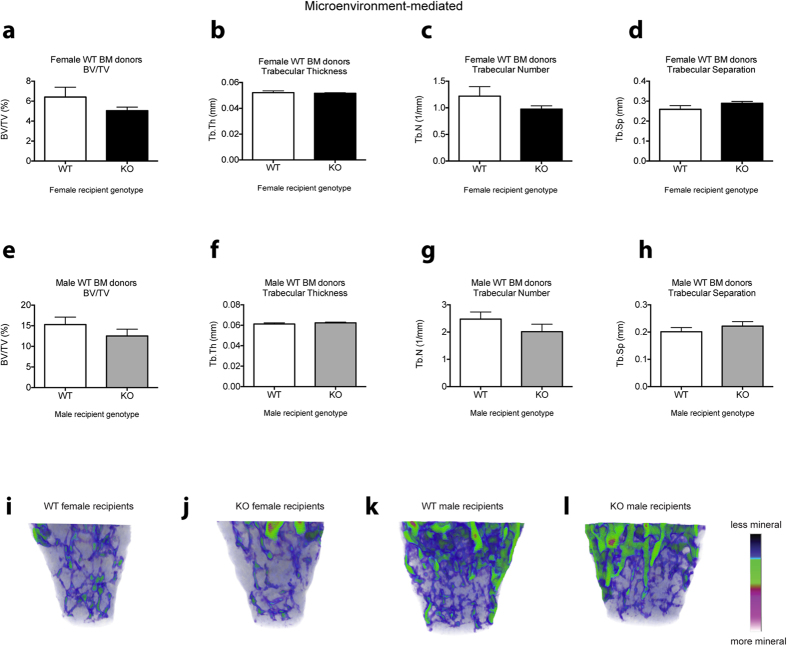
Trabecular bone phenotype in female and male *Cntf*^−/−^ and *Cntf*^+/+^ recipients of WT BM. Female (**a–d,i,j**) or male *Cntf*^−/−^ (KO) and *Cntf*^+/+^ (WT) mice (**e–h,k,l**) were transplanted with WT BM cells (female cells into female mice and male cells into male mice) and femurs were analysed using micro-computed tomography at 12 weeks post-transplantation. Shown are the trabecular bone parameters: bone volume/total volume (BV/TV) (**a,e**), trabecular thickness (**b,f**), trabecular number (**c,g**) and trabecular separation (**d,h**). Representative micro-CT images of the femurs of (**i**) female *Cntf*^+/+^ mice or (**j**) female *Cntf*^−/−^ mice transplanted with female WT BM and femurs of (**k**) male *Cntf*^+/+^ mice or (**l**) male *Cntf*^−/−^ mice transplanted with male WT BM are shown at 12 weeks post-transplant. Data are shown as mean ± SEM, n = 5–6. The unpaired Student’s T-test was used for statistical comparisons.

In contrast, when *Cntf*^+/+^ BM cells were transplanted into a *Cntf*^−/−^ microenvironment (hence measuring extrinsic regulation of haematopoiesis by CNTF), we could not observe any changes to haematopoietic parameters except for decreased spleen weights and spleen leukocyte cellularity in male *Cntf*^−/−^ recipient mice (Supplementary Fig. 6 and Fig. 6a–p). Interestingly, the increase in trabecular bone in the full *Cntf*^−/−^ females was normalised after transplantation of WT BM into female *Cntf*^−/−^ recipients (Fig. 7a–d,i,j). However, analysis of cortical bone (Supplementary Figs 6 and 9) showed that endocortical perimeter and marrow area were significantly reduced when WT BM was transplanted into female *Cntf*^−/−^ recipients compared to female *Cntf*^+/+^ recipients (Supplementary Figs 6 and 9). In contrast, there were no differences in cortical bone (Supplementary Figs 6 and 9) or trabecular bone (Fig. 7e–j,k,l) in the male *Cntf*^−/−^ transplant recipients.

## Discussion

In this study, we present data that reveal that haematopoietic-cell derived CNTF regulates haematopoiesis and bone mass. We show that, similarly to the bone phenotypes previously reported^5^ and also shown here (Fig. 2), the haematopoietic phenotype is sex-specific in the full *Cntf*^−/−^ mice. Furthermore, transplantation studies revealed that the majority of the haematopoietic and bone cell phenotypes observed in *Cntf*^−/−^ mice were due to transplantable *Cntf*^−/−^ BM cells. Interestingly, however, the B cell phenotypes observed in female WT mice transplanted with female *Cntf*^−/−^ BM cells were more striking than those observed in full *Cntf*^−/−^ female mice. Furthermore, the male WT mice transplanted with male *Cntf*^−/−^ BM cells showed a similar B lymphocyte phenotype to the female transplant recipients of female *Cntf*^−/−^ BM cells, whereas full *Cntf*^−/−^ male mice had no significant haematopoietic cell phenotypes. In addition, the trabecular bone phenotype observed in 12- and 24-week-old *Cntf*^−/−^ female mice was largely recapitulated in female WT recipients of *Cntf*^−/−^ female BM cells. Likewise, the cortical bone phenotype reported in 12-week-old full *Cntf*^−/−^ male mice was phenocopied in male WT recipients transplanted with *Cntf*^−/−^ male BM cells.

The main haematopoietic cell type affected by the loss of *Cntf* were the immature BM B220^+^IgM^+^ cells, which were significantly reduced in 12-week-old *Cntf*^−/−^ female mice and increased in 12-week-old *Cntf*^−/−^ male mice, and B220^+^IgM^−^ cells, which were significantly reduced in 24-week-old *Cntf*^−/−^ female mice. The reduction of B cells in the female mice was not due to increased death of the immature B cells since no differences were observed in apoptosis in any of the developing B cell populations in the BM, including immature B cells, in *Cntf*^−/−^ compared to *Cntf*^+/+^ female mice. Hence we focused on determining if key regulators of B cell development^13^ were perturbed in *Cntf*^−/−^ female mice. While we observed no changes in *Flt3L* or *Rankl* transcripts in whole bone marrow, there were trends to increased levels of *Cxcl12* and *Il7*, both of which have previously been shown to be expressed by osteoblast lineage cells^12^. Given that osteoblast numbers are increased in 12-week-old *Cntf*^−/−^ female mice^5^ we therefore FACS-sorted osteoblast progenitors and osteoblastic cells from these mice and their WT littermates to assess their expression of *Cxcl12* and *Il7*. We could not detect any significant differences in the expression of *Cxcl12* in osteoblast progenitors or osteoblasts from either genotype. However, *Il7* was significantly deregulated in both osteoblasts progenitors and osteoblasts, with reduced expression of *Il7* observed in *Cntf*^−/−^ osteoblast progenitors and increased expression of *Il7* in the *Cntf*^−/−^ mature osteoblasts compared to those FACS-sorted from the wildtype littermate controls.

Surprisingly, *Il7* expression was also detected in pro-B cells sorted from female *Cntf*^−/−^ mice. We cannot explain why *Il7* was increased in these cells, or if it had any functional consequences. However, it is possible that *Il7* was expressed by the pro-B cells in a compensatory manner in response to deregulated *Il7* expression by the different osteoblast lineage cells. Alternatively, it may be that *Il7* was increased in response to loss of CNTF in pro-B cells, either directly or indirectly due to increased signalling via other gp130 cytokine family members, such as IL-6. In support of this, elevated production of IL-7 has been reported in mice that have a mutation in the gp130 IL-6 receptor subunit, which results in enhanced gp130-mediated activation of signal transducer and activator of transcription 3 (STAT3)^16^.

Interestingly, IL-7 is essential for the development of pro-B and pre-B lymphocytes in the BM, and has also been shown to be important in regulating the differentiation of pre-pro-B cells into pro-B cells^17^,^18^,^19^. While we did not observe any alterations in pro-B or pre-B cells in full *Cntf*^−/−^ mice, it is interesting that pre-pro-B, pro-B, pre-B and immature B220^+^IgM^+^ cells were increased in the BM of female WT recipients transplanted with BM from female *Cntf*^−/−^ mice. Furthermore, increases in pre-pro-B and pro-B cells were observed in male WT recipients transplanted with BM from male *Cntf*^−/−^ mice, whereas in full male *Cntf*^−/−^ mice there were no B lymphocyte phenotypes, with the exception of increased B220^+^IgM^+^ cells in 12-week-old male *Cntf*^−/−^ mice. We cannot explain why the B cell phenotype is different in full *Cntf*^−/−^ mice compared to the WT recipients transplanted with *Cntf*^−/−^ BM cells. However, it is possible that loss of CNTF in other environments, including the brain, or compensatory mechanisms in the BM microenvironment, such as the altered *Il7* expression observed in the osteoblast lineage cells in *Cntf*^−/−^ female mice, may contribute to haematopoietic cell regulation in the full *Cntf*^−/−^ mice.

The differences in the haematopoietic cell phenotypes observed in the full *Cntf*^−/−^ mice compared to chimeras where the BM microenvironment was WT and the haematopoietic cells were *Cntf*^−/−^ do, however, support a role for CNTF as being a negative regulator of B lymphopoiesis. It cannot be excluded that the altered B lymphopoiesis observed in WT recipients of *Cntf*^−/−^ BM cells is regulated partly by the microenvironment since the *Cntf*^−/−^ haematopoietic cells altered the bone microenvironment in the WT recipients, and this, in turn, could de-regulate B lymphopoiesis. Indeed, the B lymphocyte phenotypes were accompanied by striking bone phenotypes, further adding to the evidence that there is a reciprocal relationship between B lymphopoiesis and the skeletal system (reviewed by Manilay and Zouali, 2014)^20^. Our data reveal that the increased trabecular bone and the reduced cortical bone parameters previously reported in 12-week-old female mice and 12-week-old male mice^5^, respectively, are also largely induced by *Cntf*^−/−^ haematopoietic cells.

In female recipients of *Cntf*^−/−^ BM cells, we observed increased trabecular number and reduced trabecular separation, but not increased bone volume. We have recently shown that bone marrow transplantation causes irreversible bone loss in mice, due to enhanced osteoclastogenesis early after the transplant^21^. This potent response to the transplant procedure likely prevented the increase in bone volume in these transplant recipients compared to non-ablated *Cntf*^−/−^ female mice. In male recipients of *Cntf*^−/−^ BM cells, the loss of cortical bone was accompanied by increased numbers of F4/80^+^CD11b^−^ pre-osteoclast cells^22^. Hence, the reduction of cortical bone observed in both the full *Cntf*^−/−^ male mice and WT male recipients transplanted with *Cntf*^−/−^ BM cells could be a result of increased osteoclast numbers and activity in the cortical region of bone.

As previously mentioned, CNTF belongs to the large IL-6 family of cytokines that all signal through the gp130 receptor. In mice, deletion of the gp130 receptor is embryonic lethal and the embryos develop defects of the haematopoietic system^23^. Haematopoietic stem and progenitor cells, T cells and megakaryocytes are severely reduced in the gp130 deficient embryos and approximately 20% of the embryos are anaemic^23^. However, when individual members of the IL-6 cytokine family such as IL-6, oncostatin M receptor (OSMR), leukaemia inhibitory factor (LIF) or CNTF are deleted, milder haematopoietic phenotypes appear. This is not surprising considering that there are likely overlapping functions of the various cytokines and in addition, the expression pattern of the different gp130 cytokines is not as broad as gp130^24^,^25^,^26^.

*Il-6*^−/−^*, Osmr*^−/−^ and *Lif*^−/−^ mice all had altered regulation of haematopoietic stem and progenitor cells, although they all exhibited different defects. *Il-6*^−/−^ mice had a mild defect where only the progenitors that give rise to colony-forming unit-spleen (CFU-S) were reduced^24^. The reduction of progenitors was more severe in *Lif*^−/−^ mice where CFU-S were severely reduced in both BM and spleen. Furthermore, other BM progenitor types such as the BFU-E and GM-CFC were reduced in *Lif*^−/−^ mice^26^. *Osmr*^−/−^ mice had reduced peripheral erythrocytes and platelets and this was reflected by reduced numbers of erythrocyte- and megakaryocyte-forming progenitors in the BM^25^. The reduction of progenitors in the BM was compensated for in the spleen where the same progenitor types were increased. In contrast to this, we did not observe any changes to the haematopoietic stem and progenitor compartment in *Cntf*^−/−^ mice, nor did we observe anaemia or thrombocytopenia in their peripheral blood.

For *Cntf*^−/−^ mice, the primary haematopoietic cell defect appears to involve B cell differentiation. This is different to the other IL-6 family members mentioned above. B cell numbers were normal in *Il-6*^−/−^*, Osmr*^−/−^ and *Lif*^−/−^ mice, although the function of mature B cells was somewhat compromised in *Il-6*^−/−^ mice. Like the *Cntf*^−/−^ mice described here, *Il-6*^−/−^*, Osmr*^−/−^ and *Lif*^−/−^ mice were all complete KOs, meaning that the defects observed could potentially arise due to changes in the microenvironment rather than due to direct effects on the haematopoietic cells. For the *Lif*^−/−^ mice it appears that lack of LIF in the microenvironment caused the haematopoietic defects^26^. The phenotypes of the *Osmr*^−/−^ mice are more complex where both direct and indirect effects on the haematopoietic cells caused the defects in erythrocyte and platelet differentiation^25^. Results from our studies of the *Cntf*^−/−^ mice suggests that CNTF has mainly intrinsic effects on haematopoiesis. In all, members of the IL-6 cytokine family, including CNTF, are important for haematopoietic cell regulation but their effects appear to be distinct to each other rather than redundant.

In conclusion, we show here that, in addition to its roles in regulating neurons and bone, CNTF has intrinsic regulatory effects on haematopoiesis, in particular B lymphopoiesis. Furthermore, we reveal, for the first time, that the bone phenotypes observed in *Cntf*^−/−^ mice occur largely due to loss of CNTF in haematopoietic cells.

## Methods

### Mice

All animal experiments were approved and conducted in accordance with the guidelines of the St. Vincent’s Health Animal Ethics Committee. *Cntf*^−/−^ mice have been previously described^4^ and are on a C57BL/6 background, their *Cntf*^+/+^ controls were generated from heterozygous matings. For bone marrow transplant experiments, WT B6.SJL-Ptprca mice (CD45.1^+^) were obtained from Animal Resources Centre, Perth, WA, Australia for use as either WT donor BM cells transplanted into *Cntf*^−/−^ or *Cntf*^+/+^ mice or as transplant recipients of *Cntf*^−/−^ or *Cntf*^+/+^ BM cells. All transplant experiments were performed using 12-week-old recipients and the mice were euthanased for analysis at 12 weeks post-transplant. For the transplants, all recipients were lethally irradiated (10 Gy γ-irradiation, split dose given three hours apart) using a Gammacell 40 Exactor (Best Theratronics, Ontario, Canada) on the day of transplant. Recipient mice were each intravenously injected with 5 × 10^6^ unfractionated BM cells obtained from the donor mice and received enrofloxacin via drinking water for 4 weeks post irradiation. All studies were performed as a minimum of two independent experiments. Mice were housed in cages and were fed with food and water *ad libitum*, with a 12 h light and 12 h dark cycle.

### Haematopoietic cell analysis

Haematopoietic cell counts from peripheral blood (PB), spleen and flushed BM were determined using a haematological analyser (Sysmex KX-21N, Roche Diagnostics). For FACS, cells obtained by pooling flushed BM and BM from crushed femurs, tibiae and ileac crests were stained with fluorescence-conjugated antibodies against murine B220, IgM, CD43, CD19, Gr-1, CD11b, F4/80, CD4 and CD8 for mature cell lineage assessment (eBioscience). Immature haematopoietic stem cell and progenitors and common lymphoid progenitors were assessed in lineage negative cells (ie. cells that did not express CD2, CD3, CD4, CD5, CD8, CD11b, Gr-1, B220 or Ter119), and positive staining with antibodies against Sca-1, c-kit and CD150 or CD127, respectively as previously described^27^. Gating strategies for some of the mature and immature haematopoietic cells are provided in Supplementary Figure 10. Apoptosis was measured in B cell populations using AnnexinV and 7AAD staining^27^. BM B cell populations were sorted using a FACSAria (BD Biosciences) or the BM, spleen, thymus and hemolysed PB cells were stained and collected for analysis using a LSRII Fortessa (BD Biosciences) with data analysed using FlowJo software version 8.8.6 (TreeStar).

### *In vitro* bone analysis

Bones were fixed in 2% paraformaldehyde (PFA, Sigma) in phosphate buffer solution (PBS) pH8.0 for 16–24 h and stored in 70% ethanol until analysed. For micro-computed tomography (micro-CT) analysis, the secondary spongiosa of the proximal femur were assessed, in accordance to standard guidelines^28^. Bones were scanned at an x-ray potential of 49 kV, a current of 100 μA and at an isotropic voxel size of 9 μm using the Skyscan1076 (Bruker-MicroCT, Kontich, Belgium). Serial tomograms were reconstructed using NRecon (Version 1.6.3.1, SkyScan) with a smoothing of 1, a ring artifact reduction of 6 and a beam hardening correction of 35%. A trabecular region 2.4 mm distal from the top of the femur extending 2 mm was analysed in CTAn (Version 1.10.1.0, SkyScan). All trabecular measurements for transplant recipients were made by manually delineating trabecular bone from the endocortical boundary, with an adaptive pre-threshold of 35–255. All trabecular measurements for 24-week-old *Cntf*^−/−^ and *Cntf*^+/+^ mice were performed using automated settings. For 24-week-old males the threshold was 46, for 24-week-old females the threshold was 31.

### Isolation of osteoblast progenitor and osteoblastic cells from bone

Osteoblast progenitor cells (lineage negative, CD45^−^, CD31^−^, CD51^+^, Sca-1^+^) and osteoblastic cells (lineage negative, CD45^−^, CD31^−^, CD51^+^, Sca-1^−^) were sorted from collagenase-digested long bones obtained from 12-week-old female *Cntf*^−/−^ and *Cntf*^+/+^ mice as previously described^11^,^15^. All antibodies were obtained from eBioscience.

### Quantitative real-time PCR analysis (qPCR)

Quantitative real-time PCR (qPCR) was performed on cDNA using Brilliant II Sybr Green QPCR master mix (Agilent Technologies) on the MX3000P Multiplex Quantitative PCR system (Stratagene) with oligonucleotide primers (IDT). Primers for *Rankl* and housekeeping genes have been previously published^21^. Primers for *Il7* are also published^29^. Primer sequences are as follows: *Cxcl12*: Forward: GAG CCA ACG TCA AGC ATC TG; Reverse: GAG CCA ACG TCA AGC ATC TG. *Flt3-L*: Forward: CCC AGC CAG TCA GCG TTG GT; Reverse: CAC GTG CAT CGG ATT CGT GGG T.

### Statistical analysis

One-way analysis of variance followed by post-hoc testing was used for multiple comparisons. The unpaired Student’s T-test was used for statistical comparisons between two different populations. All statistical analysis was performed using Prism 6 software (GraphPad).

## Additional Information

**How to cite this article**: Askmyr, M. *et al.* Ciliary neurotrophic factor has intrinsic and extrinsic roles in regulating B cell differentiation and bone structure. *Sci. Rep.* **5**, 15529; doi: 10.1038/srep15529 (2015).

## Supplementary Material

Supplementary Information

## References

[b1] Sims N. A. & Walsh N. C. GP130 cytokines and bone remodelling in health and disease. BMB Reports. 43, 513–523 (2010). 20797312 10.5483/bmbrep.2010.43.8.513

[b2] Wagener E. M. *et al.* The amino acid exchange R28E in ciliary neurotrophic factor (CNTF) abrogates interleukin-6 receptor-dependent but retains CNTF receptor-dependent signaling via glycoprotein 130 (gp130)/leukemia inhibitory factor receptor (LIFR). J. Biol. Chem. 289, 18442–18450, doi: 10.1074/jbc.M114.568857 (2014). 24802752 PMC4140248

[b3] DeChiara T. M. *et al.* Mice lacking the CNTF receptor, unlike mice lacking CNTF, exhibit profound motor neuron deficits at birth. Cell. 83, 313–322 (1995). 7585948 10.1016/0092-8674(95)90172-8

[b4] Masu Y. *et al.* Disruption of the CNTF gene results in motor neuron degeneration. Nature. 365, 27–32, doi: 10.1038/365027a0 (1993). 8361533

[b5] McGregor N. E. *et al.* Ciliary neurotrophic factor inhibits bone formation and plays a sex-specific role in bone growth and remodeling. Calcif. Tissue Int. 86, 261–270, doi: 10.1007/s00223-010-9337-4 (2010). 20157807

[b6] Askmyr M., Sims N. A., Martin T. J. & Purton L. E. What is the true nature of the osteoblastic hematopoietic stem cell niche? Trends Endocrinol. Metab. 20, 303–309, doi: 10.1016/j.tem.2009.03.004 (2009). 19595609

[b7] Calvi L. M. *et al.* Osteoblastic cells regulate the haematopoietic stem cell niche. Nature. 425, 841–846, doi: 10.1038/nature02040 (2003). 14574413

[b8] Zhang J. *et al.* Identification of the haematopoietic stem cell niche and control of the niche size. Nature. 425, 836–841 (2003). 14574412 10.1038/nature02041

[b9] Tokoyoda K., Egawa T., Sugiyama T., Choi B. I. & Nagasawa T. Cellular niches controlling B lymphocyte behavior within bone marrow during development. Immunity. 20, 707–718, doi: 10.1016/j.immuni.2004.05.001 (2004). 15189736

[b10] Zhu J. *et al.* Osteoblasts support B-lymphocyte commitment and differentiation from hematopoietic stem cells. Blood. 109, 3706–3712, doi: 10.1182/blood-2006-08-041384 (2007). 17227831

[b11] Visnjic D. *et al.* Hematopoiesis is severely altered in mice with an induced osteoblast deficiency. Blood. 103, 3258–3264, doi: 10.1182/blood-2003-11-4011 (2004). 14726388

[b12] Wu J. Y. *et al.* Osteoblastic regulation of B lymphopoiesis is mediated by Gs{alpha}-dependent signaling pathways. Proc. Natl. Acad. Sci. USA 105, 16976–16981, doi: 10.1073/pnas.0802898105 (2008). 18957542 PMC2579363

[b13] Nagasawa T. Microenvironmental niches in the bone marrow required for B-cell development. Nat. Rev. Immunol. 6, 107–116, doi: 10.1038/nri1780 (2006). 16491135

[b14] Semerad C. L. *et al.* G-CSF potently inhibits osteoblast activity and CXCL12 mRNA expression in the bone marrow. Blood. 106, 3020–3027, doi: 10.1182/blood-2004-01-0272 (2005). 16037394 PMC1895331

[b15] Noll J. E. *et al.* Myeloma plasma cells alter the bone marrow microenvironment by stimulating the proliferation of mesenchymal stromal cells. Haematologica. 99, 163–171, doi: 10.3324/haematol.2013.090977 (2014). 23935020 PMC4007935

[b16] Sawa S. *et al.* Autoimmune arthritis associated with mutated interleukin (IL)-6 receptor gp130 is driven by STAT3/IL-7-dependent homeostatic proliferation of CD4+ T cells. J. Exp. Med. 203, 1459–1470, doi: 10.1084/jem.20052187 (2006). 16717113 PMC2118324

[b17] Peschon J. J. *et al.* Early lymphocyte expansion is severely impaired in interleukin 7 receptor-deficient mice. J. Exp. Med. 180, 1955–1960 (1994). 7964471 10.1084/jem.180.5.1955 PMC2191751

[b18] von Freeden-Jeffry U. *et al.* Lymphopenia in interleukin (IL)-7 gene-deleted mice identifies IL-7 as a nonredundant cytokine. J. Exp. Med. 181, 1519–1526 (1995). 7699333 10.1084/jem.181.4.1519 PMC2191954

[b19] Dias S., Silva H. Jr., Cumano A. & Vieira P. Interleukin-7 is necessary to maintain the B cell potential in common lymphoid progenitors. J. Exp. Med. 201, 971–979, doi: 10.1084/jem.20042393 (2005). 15767371 PMC2213099

[b20] Manilay J. O. & Zouali M. Tight relationships between B lymphocytes and the skeletal system. Trends Mol. Med. 20, 405–412, doi: 10.1016/j.molmed.2014.03.003 (2014). 24726716

[b21] Quach J. M. *et al.* Myelosuppressive therapies significantly increase pro-inflammatory cytokines and directly cause bone loss. J. Bone Miner. Res. doi: 10.1002/jbmr.2415 (2014). 25418357

[b22] Takeshita S., Kaji K. & Kudo A. Identification and characterization of the new osteoclast progenitor with macrophage phenotypes being able to differentiate into mature osteoclasts. J. Bone Miner. Res. 15, 1477–1488, doi: 10.1359/jbmr.2000.15.8.1477 (2000). 10934646

[b23] Yoshida K. *et al.* Targeted disruption of gp130, a common signal transducer for the interleukin 6 family of cytokines, leads to myocardial and hematological disorders. Proc. Natl. Acad. Sci. USA 93, 407–411 (1996). 8552649 10.1073/pnas.93.1.407 PMC40247

[b24] Kopf M. *et al.* Pleiotropic defects of IL-6-deficient mice including early hematopoiesis, T and B cell function, and acute phase responses. Ann. NY Acad. Sci. 762, 308–318 (1995). 7545368 10.1111/j.1749-6632.1995.tb32335.x

[b25] Tanaka M. *et al.* Targeted disruption of oncostatin M receptor results in altered hematopoiesis. Blood. 102, 3154–3162, doi: 10.1182/blood-2003-02-0367 (2003). 12855584

[b26] Escary J. L., Perreau J., Dumenil D., Ezine S. & Brulet P. Leukaemia inhibitory factor is necessary for maintenance of haematopoietic stem cells and thymocyte stimulation. Nature. 363, 361–364, doi: 10.1038/363361a0 (1993). 8497320

[b27] Purton L. E. *et al.* RARgamma is critical for maintaining a balance between hematopoietic stem cell self-renewal and differentiation. J. Exp. Med. 203, 1283–1293, doi: 10.1084/jem.20052105 (2006). 16682494 PMC2121209

[b28] Bouxsein M. L. *et al.* Guidelines for assessment of bone microstructure in rodents using micro-computed tomography. J. Bone Miner. Res. 25, 1468–1486, doi: 10.1002/jbmr.141 (2010). 20533309

[b29] Green A. C. *et al.* RARgamma is a negative regulator of osteoclastogenesis. J. Steroid Biochem. Mol. Biol. 150, 46–53, doi: 10.1016/j.jsbmb.2015.03.005 (2015). 25800721

