# Supplementary material for: Ciliary neurotrophic factor has intrinsic and extrinsic roles in regulating B cell differentiation and bone structure
Source: Sci Rep. 2015 Oct 21;5:15529. doi: 10.1038/srep15529 (PMC4614391; doi:10.1038/srep15529)
Supplement: Supplementary Information [file srep15529-s1.pdf]

Supplementary Information

Ciliary neurotrophic factor has intrinsic and extrinsic roles in regulating B cell differentiation and bone structure.

Maria Askmyr[1,2,+], Kirby White[1,+], Tanja Jovic[1], Hannah A. King[1], Julie M. Quach[1], Ana C. Maluenda[1], Emma K. Baker[1,3], Monique F. Smeets[1], Carl R. Walkley[1,3] and Louise E. Purton[1,3,*]

[1]Stem Cell Regulation Unit, St. Vincent's Institute of Medical Research, Fitzroy, Vic. 3065, Australia

[2]Department of Clinical Genetics, Lund University, Lund, Sweden,

[3] Department of Medicine at St. Vincent's Hospital, The University of Melbourne, Fitzroy, Vic. 3065, Australia

+ These authors contributed equally

***Corresponding author:**

Louise Purton, PhD

Address: Stem Cell Regulation Unit, St. Vincent's Institute of Medical Research, 9 Princes Street, Fitzroy, Vic., 3065, Australia

Phone: +61 3 9231 2504

Fax: +61 3 9416 2676

E-mail: lpurton@svi.edu.au

Supplementary Figure 1

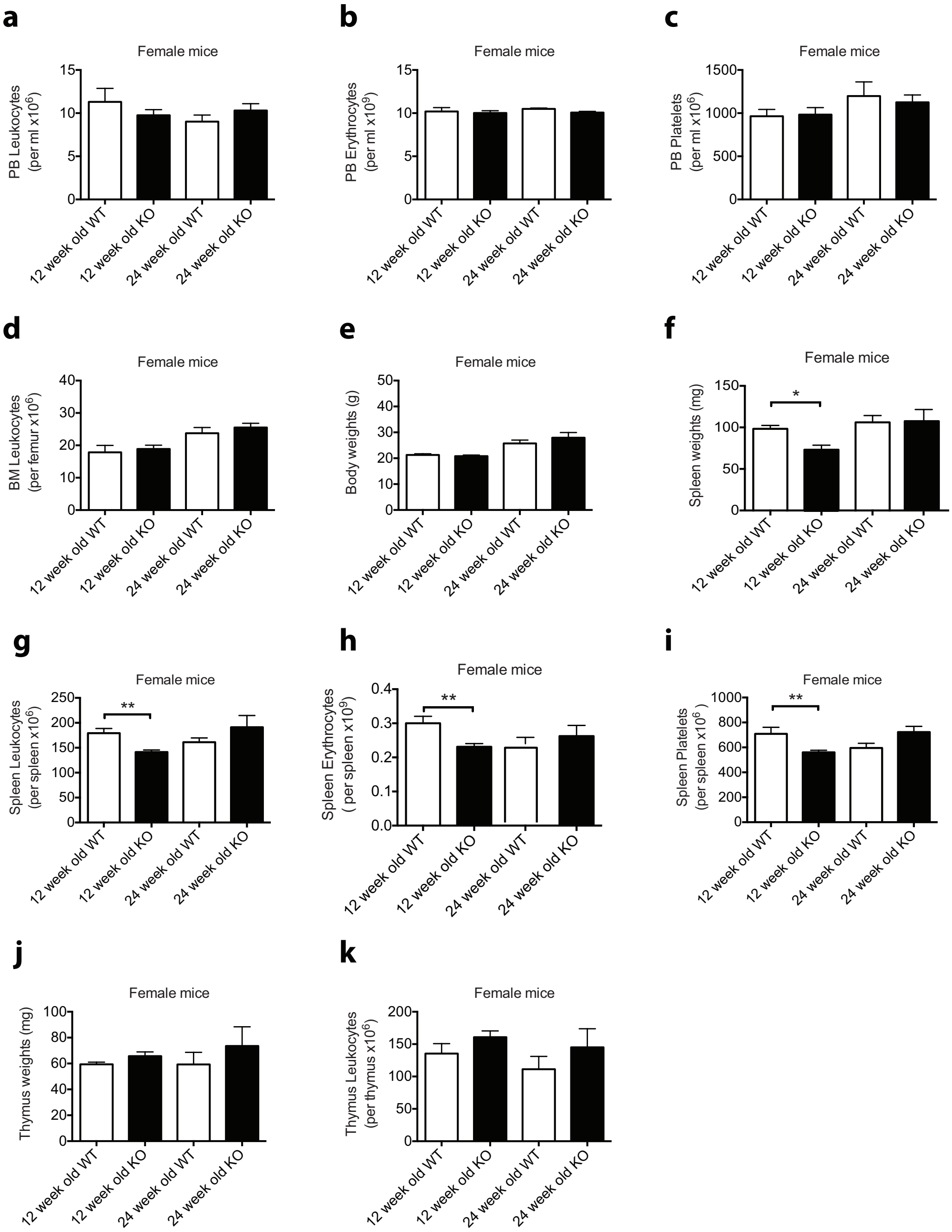

Supplementary Figure S1. Haematopoietic cell content in PB, BM and spleen of female *Cntf*$^{-/-}$ and *Cntf*$^{+/+}$ mice.

Haematopoietic cell numbers (PB, BM, spleen and thymus) and weights of total body, spleen and thymus was analysed and are presented here as follows: PB leukocytes (a), PB erythrocytes (b), PB platelets, BM leukocytes (d), body weights (e), spleen weights (f), spleen leukocytes (g), spleen erythrocytes (h), spleen platelets (i), thymus weights (j) and thymus leukocytes (k). Data are shown as mean ± SEM, n= 6-9. The unpaired Student's T-test was used for comparisons between age-matched mice. *P<0.05, **P<0.01.

Supplementary Figure 2

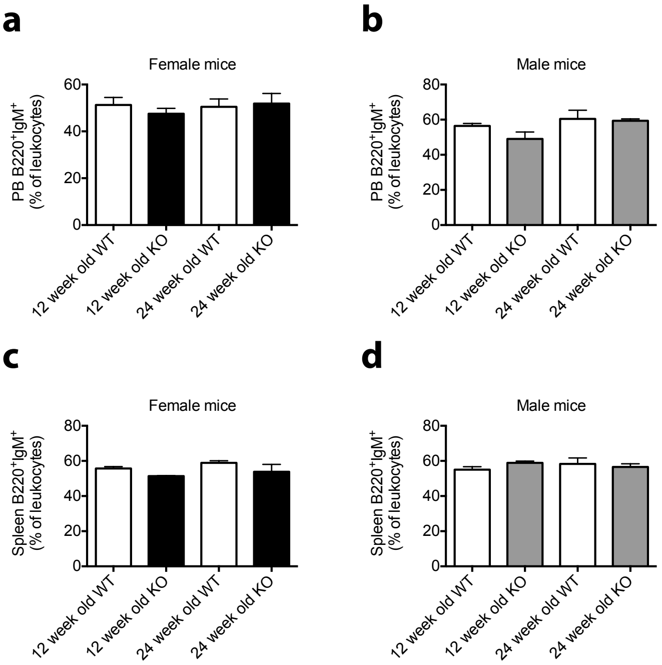

Supplementary Figure S2. Analysis of PB and spleen B cell populations in female and male *Cntf*$^{-/-}$ and *Cntf*$^{+/+}$ mice.

Analysis of PB (a and b) and spleen (c and d) B220^{+}IgM^{+} immature B cells in 12 and 24-week-old female (a and c) and male (b and d) *Cntf*$^{-/-}$ (KO) and *Cntf*$^{+/+}$ (WT) mice. Data are shown as mean ± SEM, n= 6-9. The unpaired Student's T-test was used for comparisons between age-matched mice.

Supplementary Figure 3

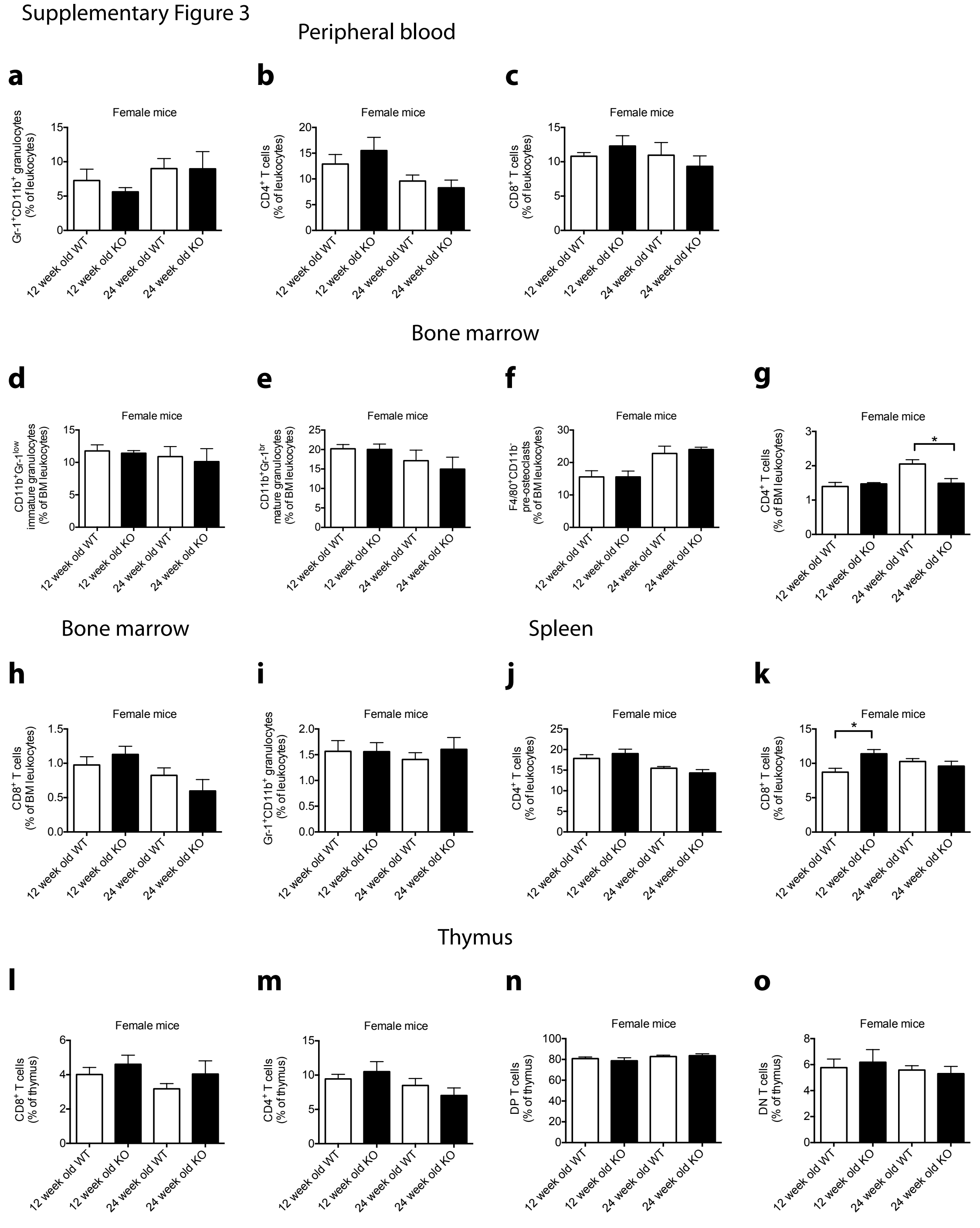

Supplementary Figure S3. Haematopoietic lineage analysis of PB, BM, spleen and thymus of *Cntf*$^{-/-}$ and *Cntf*$^{+/+}$ female mice.

Lineage analysis of PB, BM, spleen and thymus in *Cntf*$^{-/-}$ (KO) and *Cntf*$^{+/+}$ (WT) 12 and 24-week-old female mice. Panels a-c show PB Gr-1^{+}CD11b^{+} granulocytes (a), CD4^{+} T cells (b) and CD8^{+} T cells (c). Panels d-h show BM CD11b^{+}Gr-1^{low} immature granulocytes (d), CD11b^{+}Gr-1^{br} mature granulocytes (e), F4/80^{+}CD11b^{-} pre-osteoclasts (f), CD4^{+} T cells (g) and CD8^{+} T cells (h). Panels i-k show spleen Gr-1^{+}CD11b^{+} granulocytes (i), CD4^{+} T cells (j) and CD8^{+} T cells (k). Panels l-o show thymus CD8^{+} T cells (l), CD4^{+} T cells (m), double positive (DP) T cells (n) and double negative (DN) T cells (o). Data are shown as mean ± SEM, n= 4-6. The unpaired Student's T-test was used for comparisons between age-matched mice. *$P<0.05$, **$P<0.01$.

Supplementary Figure 4

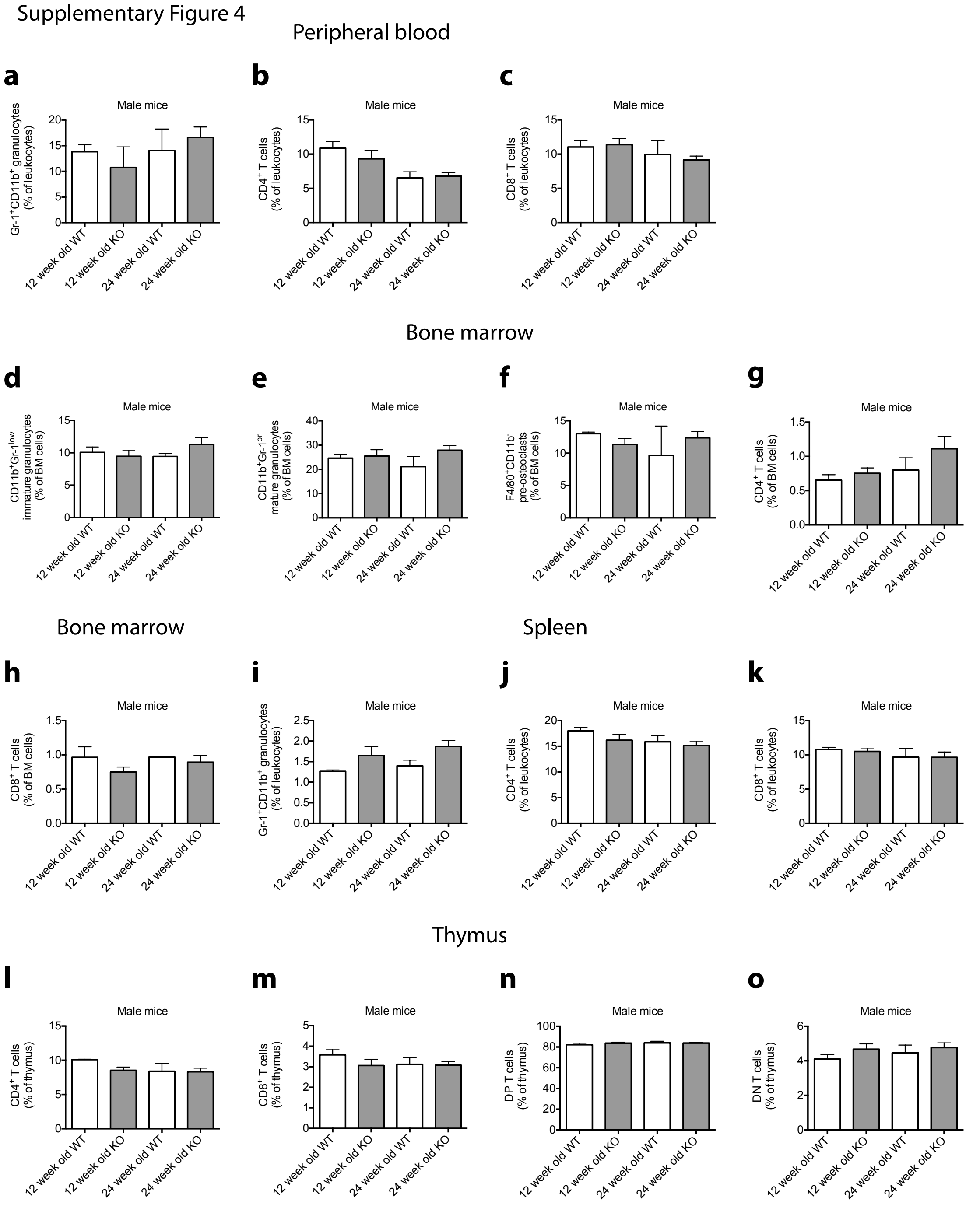

Supplementary Figure S4. Haematopoietic lineage analysis of PB, BM, spleen and thymus of *Cntf*$^{-/-}$ and *Cntf*$^{+/+}$ male mice.

Lineage analysis of PB, BM, spleen and thymus in *Cntf*$^{-/-}$ (KO) and *Cntf*$^{+/+}$ (WT) 12 and 24-week-old male mice. Panels a-c show PB Gr-1^{+}CD11b^{+} granulocytes (a), CD4^{+} T cells (b) and CD8^{+} T cells (c). Panels d-h show BM CD11b^{+}Gr-1^{low} immature granulocytes (d), CD11b^{+}Gr-1^{br} mature granulocytes (e), F4/80^{+}CD11b^{-} pre-osteoclasts (f), CD4^{+} T cells (g) and CD8^{+} T cells (h). Panels i-k show spleen Gr-1^{+}CD11b^{+} granulocytes (i), CD4^{+} T cells (j) and CD8^{+} T cells (k). Panels l-o show thymus CD4^{+} T cells (l), CD8^{+} T cells (m), double positive (DP) T cells (n) and double negative (DN) T cells (o). Data are shown as mean ± SEM, n= 3-7. The unpaired Student's T-test was used for comparisons between age-matched mice.

Supplementary Figure 5

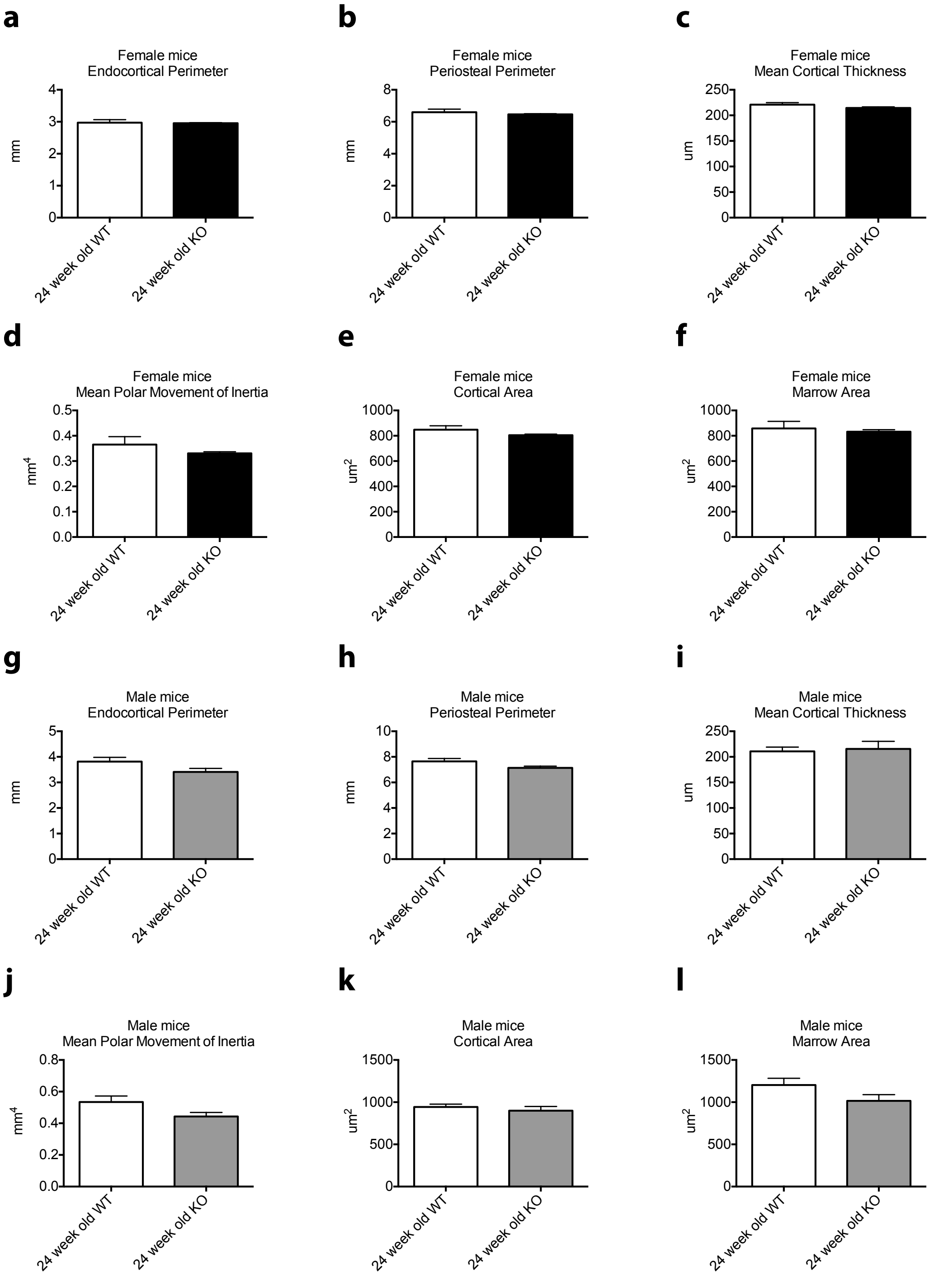

Supplementary Figure S5. Cortical bone phenotype in 24-week-old female and male *Cntf*$^{-/-}$ and *Cntf*$^{+/+}$ mice.

The cortical bone phenotype was analysed in 24-week-old female (a-f) and male (g-l) *Cntf*$^{-/-}$ (KO) and *Cntf*$^{+/+}$ (WT) mice and are presented here as: endocortical perimeter (a and g), periosteal perimeter (b and h), mean cortical thickness (c and i), mean polar movement of inertia (d and j), cortical area (e and k) and marrow area (f and l). Data are shown as mean ± SEM, n= 4-6. The unpaired Student's T-test was used for statistical comparisons.

Supplementary Figure 6

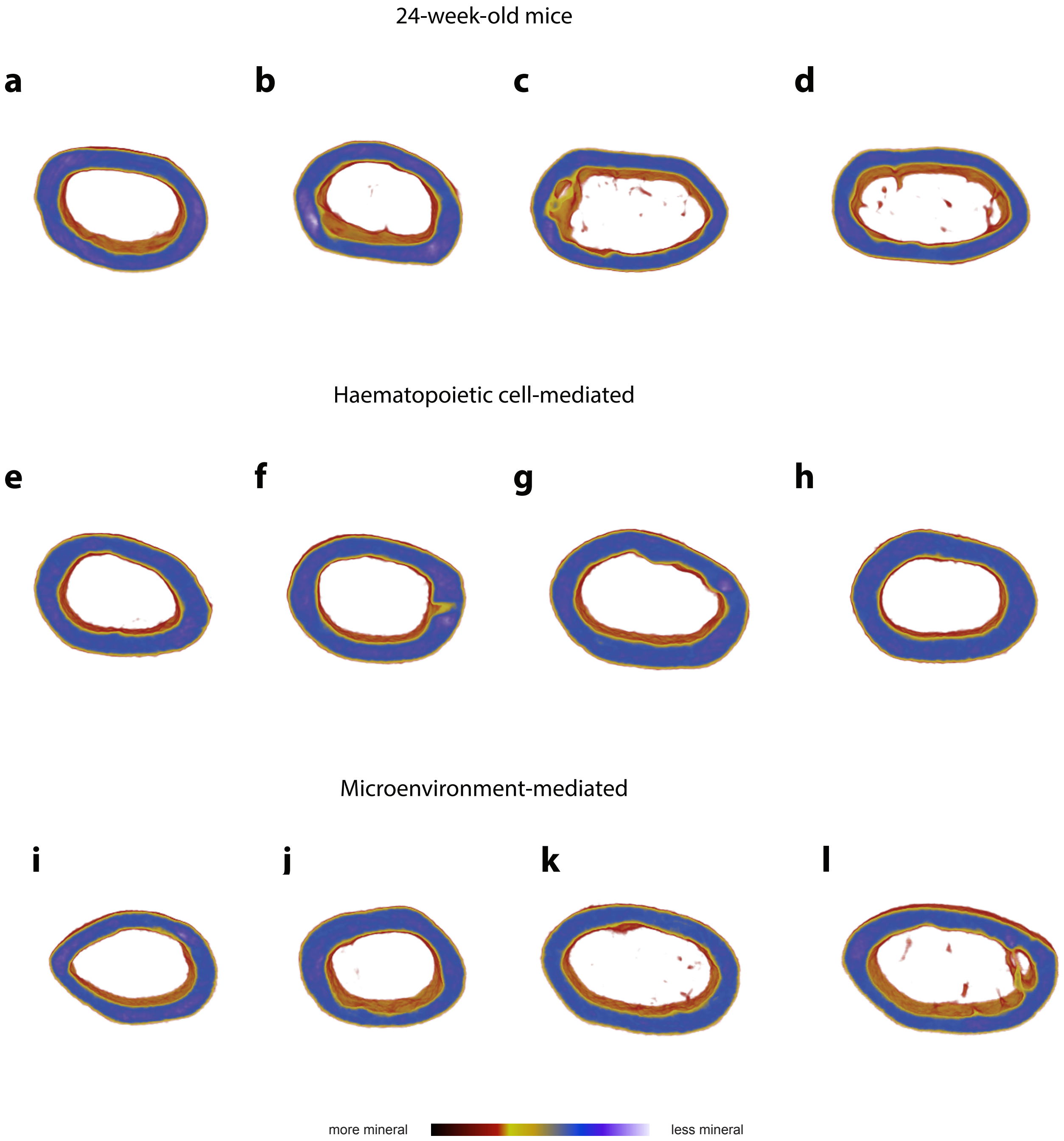

Supplementary Figure S6. Representative micro-CT pictures of cortical bone analyses of the mice. Shown are pictures from (a) a 24-week-old *Cntf*+/+ female mouse, (b) a 24-week-old *Cntf*-/- female mouse, (c) a 24-week-old *Cntf*+/+ male mouse, (d) a 24-week-old *Cntf*-/- male mouse, (e) a female WT recipient transplanted with female *Cntf*+/+ BM cells, (f) a female WT recipient transplanted with female *Cntf*-/- BM cells, (g) a male WT recipient transplanted with male *Cntf*+/+ BM cells, (h) a male WT recipient transplanted with male *Cntf*-/- BM cells, (i) a female *Cntf*+/+ recipient transplanted with female WT BM cells, (j) a female *Cntf*-/- recipient transplanted with female WT BM cells, (k) a male *Cntf*+/+ recipient transplanted with male WT BM cells and (l) a male *Cntf*-/- recipient transplanted with male WT BM cells.

Supplementary Figure 7

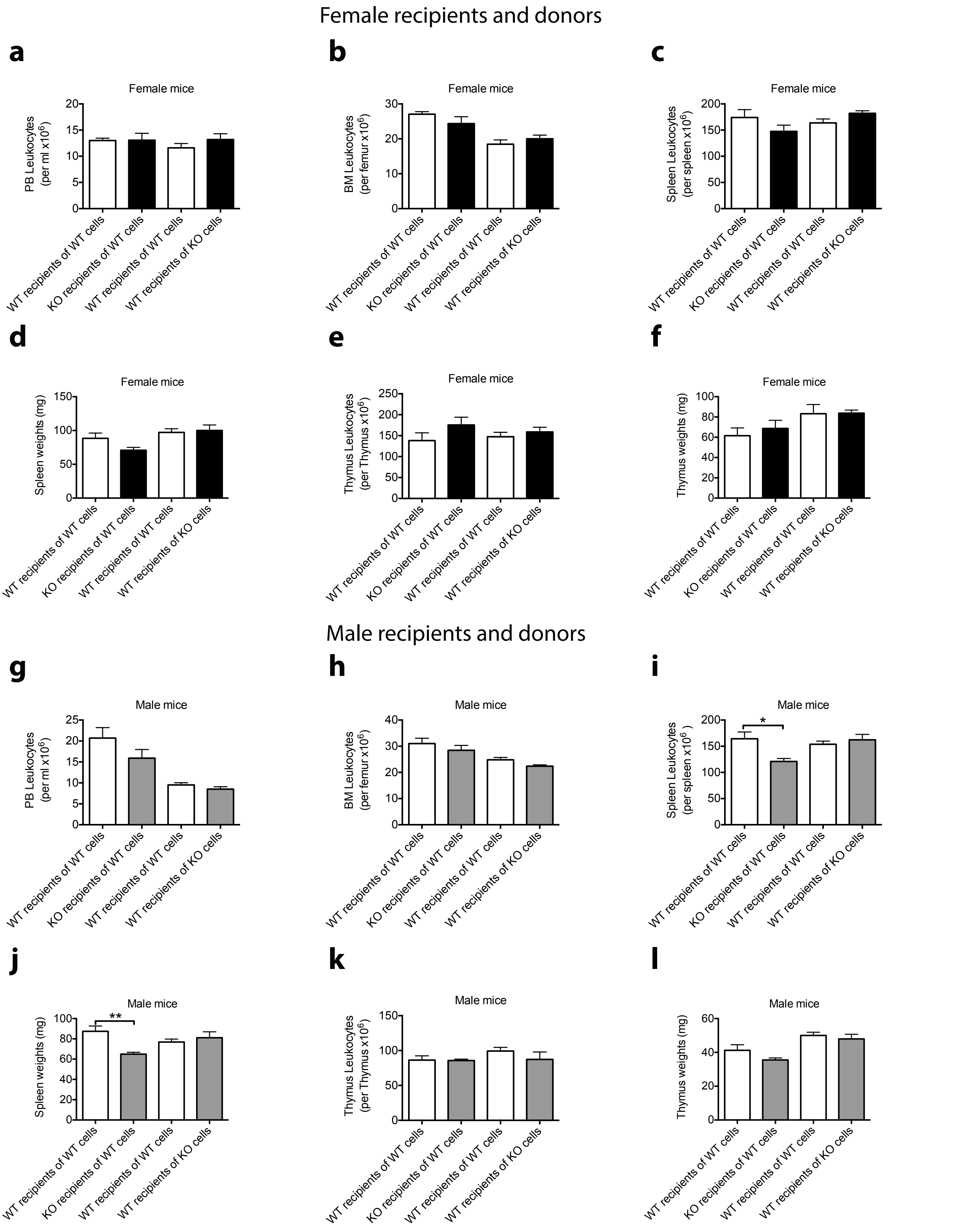

Supplementary Figure S7. Haematopoietic content of transplanted WT, *Cntf*+/+ or *Cntf-/-* female and male mice. Haematopoietic cell content of PB, BM, spleen and thymus and weights of spleen and thymus were analysed in transplanted female (a-f) and male (g-l) mice. Mice were either WT recipients transplanted with *Cntf*+/+ (WT) or *Cntf*-/- (KO) BM cells, or *Cntf*+/+ (WT) or *Cntf*-/- (KO) recipients transplanted with WT BM cells. The following parameters were analysed: PB leukocytes (a and g), BM leukocytes (b and h), spleen leukocytes (c and l), spleen weights (d and j), thymus leukocytes (e and k) and thymus weights (f-l). Data are shown as mean ± SEM, n= 5-10. The unpaired Student's t-test was used for statistical comparisons of each transplant type and sex. *P<0.05, **P<0.01.

Supplementary Figure 8

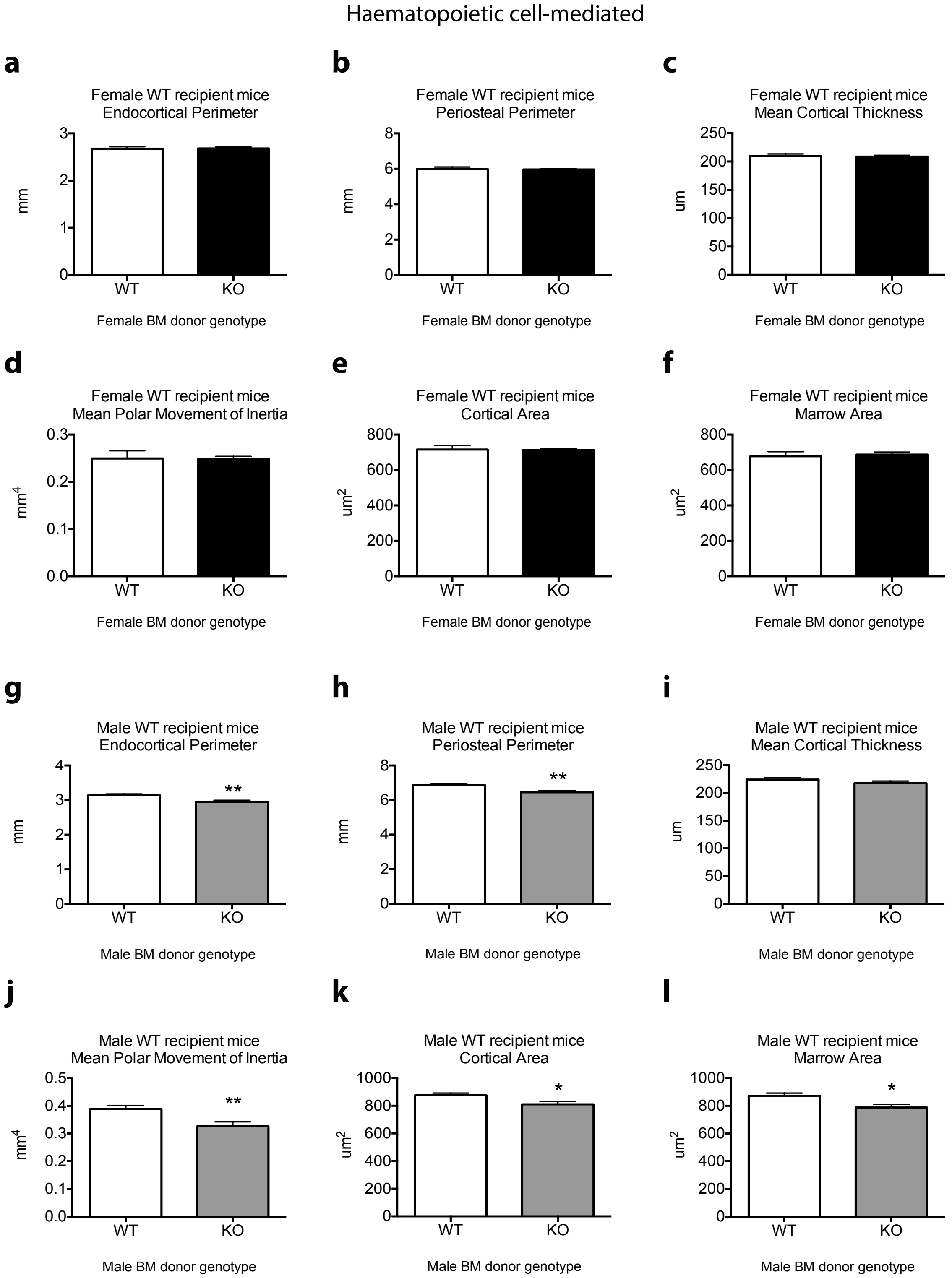

Supplementary Figure S8. Cortical bone phenotypes in transplanted female and male WT recipients of *Cntf*+/+ or *Cntf*-/- BM cells. The cortical bone phenotypes were analysed in WT recipients of *Cntf*+/+ (WT) or C*ntf*-/- (KO) BM at 12 weeks post-transplant. Data for female WT recipients are shown in (a-f), data for male WT recipients are shown in (g-l). The following parameters were analysed: endocortical perimeter (a and g), periosteal perimeter (b and h), mean cortical thickness (c and i), mean polar movement of intertia (d and j), cortical area (e and k) and marrow area (f and l). Data are shown as mean ± SEM, n= 5-10. The unpaired Student's T-test was used for statistical comparisons. **P*<0.05, ***P*<0.01.

Supplementary Figure 9

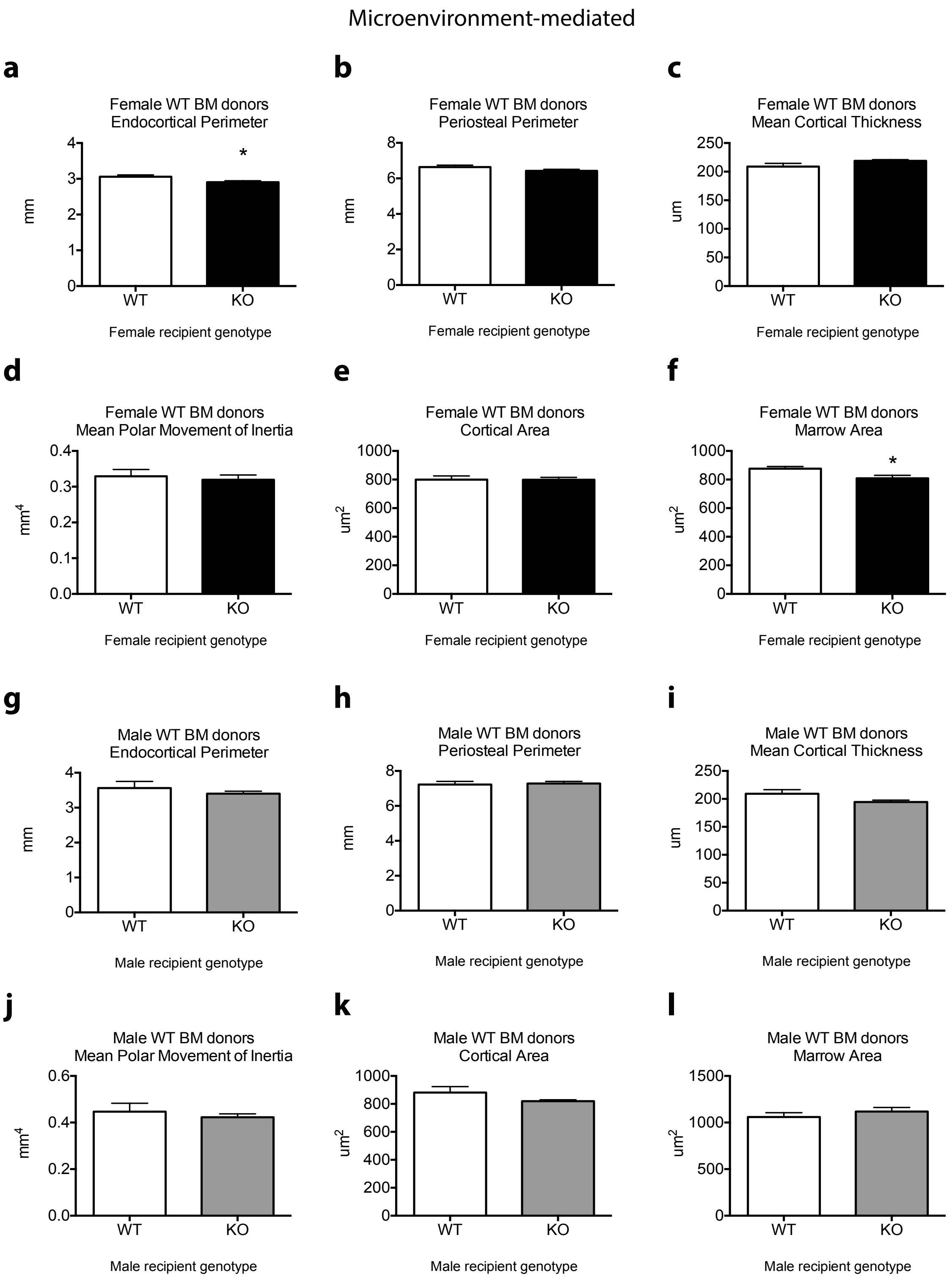

Supplementary Figure S9. Cortical bone phenotypes in transplanted female and male *Cntf-/-* and *Cntf+/+* recipients of WT BM cells. The cortical bone phenotypes were analysed in *Cntf-/-* (KO) or *Cntf+/+* (WT) recipients of WT BM at 12 weeks post-transplant. Data for female *Cntf* KO and WT recipients are shown in (a-f), data for male *Cntf* KO and WT recipients are shown in (g-l). The following parameters were analysed: endocortical perimeter (a and g), periosteal perimeter (b and h), mean cortical thickness (c and i), mean polar movement of intertia (d and j), cortical area (e and k) and marrow area (f and l). Data are shown as mean ± SEM, n= 5-6. The unpaired Student's T-test was used for statistical comparisons. *P<0.05.

Supplementary Figure 10

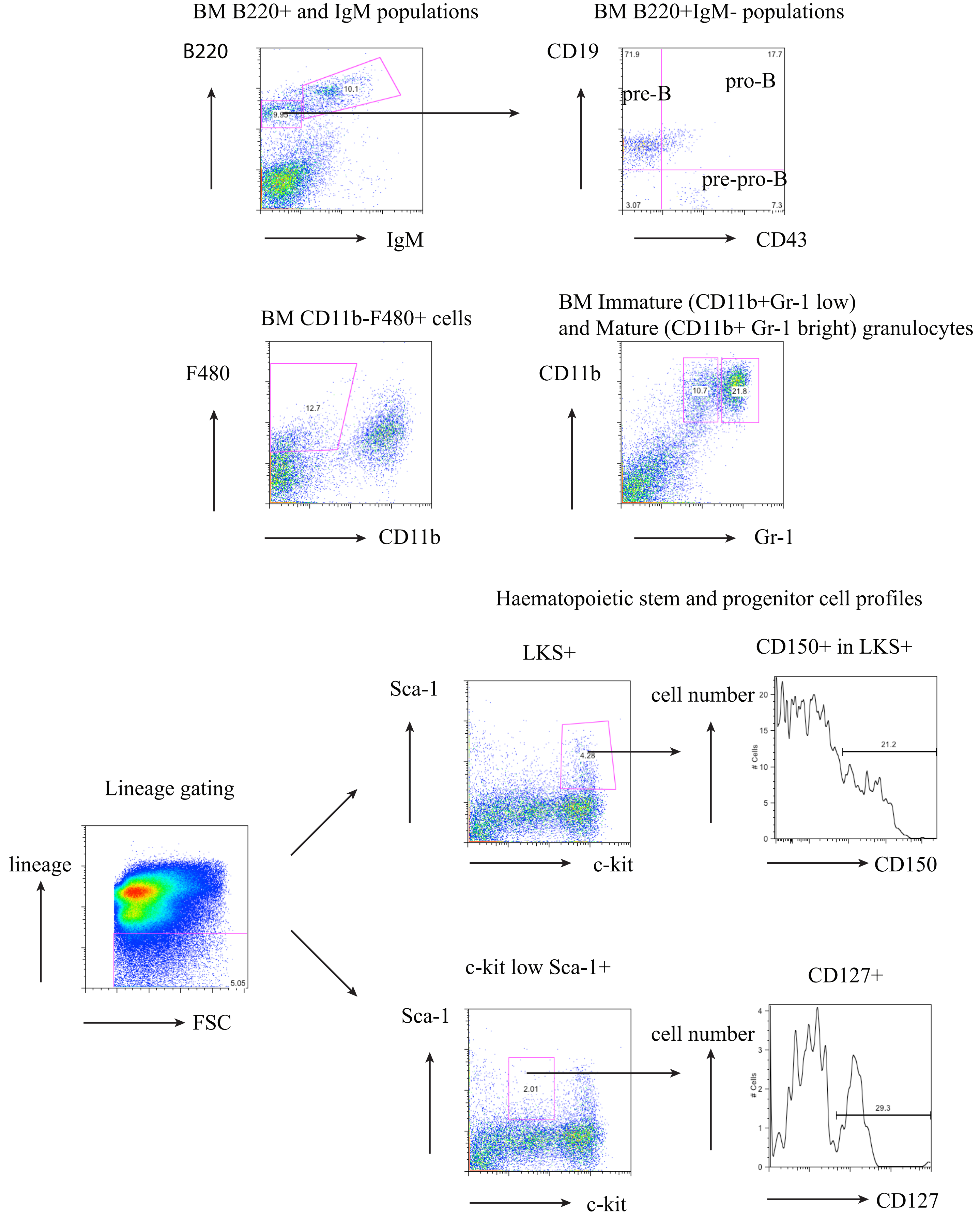

Supplementary Figure S10. Representative FACS plots of BM cell populations altered in different CNTF WT and KO experiments, plus gating strategies for haematopoietic stem and progenitor cells. FSC= forward scatter. LKS+= lineage negative, c-kit+, Sca-1+.
